# Targeting AhR as a Novel Therapeutic Modality against Inflammatory Diseases

**DOI:** 10.3390/ijms23010288

**Published:** 2021-12-28

**Authors:** Alkeiver S. Cannon, Prakash S. Nagarkatti, Mitzi Nagarkatti

**Affiliations:** Department of Pathology, Microbiology, and Immunology, University of South Carolina School of Medicine, Columbia, SC 29209, USA; alkeiver.cannon@uscmed.sc.edu (A.S.C.); prakash@mailbox.sc.edu (P.S.N.)

**Keywords:** aryl hydrocarbon receptor, TCDD, inflammation, inflammatory disease, epigenetic, multiple sclerosis, inflammatory bowel diseases, atopic dermatitis

## Abstract

For decades, activation of Aryl Hydrocarbon Receptor (AhR) was excluded from consideration as a therapeutic approach due to the potential toxic effects of AhR ligands and the induction of the cytochrome P450 enzyme, Cyp1a1, following AhR activation. However, it is now understood that AhR activation not only serves as an environmental sensor that regulates the effects of environmental toxins, but also as a key immunomodulator where ligands induce a variety of cellular and epigenetic mechanisms to attenuate inflammation. Thus, the emergence of further in-depth research into diverse groups of compounds capable of activating this receptor has prompted reconsideration of its use therapeutically. The aim of this review is to summarize the body of research surrounding AhR and its role in regulating inflammation. Specifically, evidence supporting the potential of targeting this receptor to modulate the immune response in inflammatory and autoimmune diseases will be highlighted. Additionally, the opportunities and challenges of developing AhR-based therapies to suppress inflammation will be discussed.

## 1. Introduction

Inflammatory and autoimmune disease development is significantly affected by a number of factors including environmental pollutants, the microbiome, diet, and metabolism [1]. It is known that regulation of the immune system in such diseases involves both genetic and environmental factors, though much is still unknown regarding the latter. These factors can be integrated through the AhR, a ligand-dependent transcription factor that controls various transcriptional processes. Though this receptor is historically known for its function as an environmental sensor, attention has shifted towards elucidating its role in innate and adaptive immune functions and as a possible therapeutic target, partly due to its expression in many vertebrate cells including numerous immune cell types and barrier organs such as the skin, intestine, and lung [2,3]. In this review, we focus on the source of AhR ligands, the mechanisms and immunological changes through which they attenuate inflammation, and diseases where their use as a treatment has been shown to be beneficial. We also discuss the role of the microbiota and AhR mutations in triggering inflammation which, when taken together, lend credence to the potential of AhR as a therapeutic target.

## 2. Introduction to Inflammation

Inflammation is characterized as an adaptive response to harmful stimuli and conditions aimed at removing the causative agent and returning the host to homeostasis [4]. It is the underlying component of many processes and, when adequately regulated, can act as an efficient protector against infection and wound healing [5]. Dysregulation of inflammation, however, can lead to excessive tissue damage and increased risk of chronic diseases [4,6].

Tissue injury and infection are conventional causes of inflammation that induce either an acute or chronic response [6]. If the causative agent can be cleared relatively quickly, the host’s immune system will launch an acute inflammatory response. During this type of response, the goal is to get white blood cells and plasma proteins to the site of injury. To accomplish this, vascular permeability is increased which allows for extravasation of leukocytes [7]. Resident immune cells, such as mast cells, macrophages, and dendritic cells typically sense this type of inflammation via innate pathogen recognition receptors (PRRs), including toll-like receptors (TLRs) and nucleotide-binding oligomerization domain (NOD)-like receptors, and induce the synthesis of cytokines leading to the activation of downstream proinflammatory pathways [8,9,10]. Specifically, the TLR signaling pathway has been implicated in rheumatoid arthritis (RA) in that TLRs were increased in multiple immune cell types [11,12] as TLR-2 and TLR-4 ligand responses were increased in RA patients [13]. Pro-inflammatory cytokines, such as tumor necrosis factor (TNF), interleukin (IL)-1, and IL-6, direct the inflammatory response by regulating activities, such as the recruitment of blood cells and endothelial permeability [7]. TNF-alpha specifically has been identified as a therapeutic target for RA due to its role in activating macrophages and lymphocytes and increasing secretion of other inflammatory cytokines [14]. The acute response is over once the initiator has been removed and the affected tissue has been repaired [15]. Throughout this process, damage to the host tissue is inevitable [4,6].

If the inflammatory inducer is unable to be cleared swiftly, the inflammation will continue into a more chronic state. While impairment of the tissue, organ, or organ system often causes chronic inflammation, autoimmune responses are sometimes the culprit, and thus lead to autoimmune diseases [4]. In autoimmune diseases, the damage is caused by self-reactive, adaptive immune responses caused by a loss of tolerance [16,17]. Bo et al. showed that this loss of tolerance could be triggered by viral and bacterial infections such as those caused by Epstein–Barr virus (EBV) and *Mycobacterium avium* subspecies *paratuberculosis* (MAP) [18]. In these studies, interferon regulatory factor 5 (IRF5) was identified as an autoimmune target of RA due to increased reactivity to IRF5 antibodies in sera samples from RA patients [19]. In the case of autoimmune diseases, self-antigens are the target, however, because removal of them is not possible, this state of chronic inflammation usually leads to further tissue damage [20].

The environment has been proposed as a possible player in autoimmune disorders due to increasing rates of these diseases in industrialized countries [16]. Other culprits include human endogenous retroviruses K/W (HERV-W/K), species of *Mycobacteria*, and EBV as triggers of multiple sclerosis (MS) [21,22], neuromyelitis optica spectrum disorder (NMOSD) [21,23], amyotrophic lateral sclerosis (ALS) [24,25], diabetes [26], and RA [27]. While progress has been made in terms of treatments, many of these conditions progress slowly so tissue damage may have occurred before the diseases are diagnosed [16]. Additionally, some of the agents used are overtly immunosuppressive thereby increasing the chances of the patients’ acquiring infections and cancer [28]. Thus, it is necessary to investigate therapeutic targets that can prevent and treat inflammatory and autoimmune diseases including the potential to reverse the damage caused by inflammation. Studies support the notion that these goals can be accomplished with targeting AhR.

## 3. Introduction to AhR

Depending on the cells, tissues, and organs expressing AhR, the role it plays in the inflammatory response may vary. This receptor is a member of the basic helix-loop-helix (bHLH) transcription factor superfamily and contains a central periodic circadian protein (PER)-AhR nuclear translator (ARNT)-single-minded (SIM) protein domain [29]. The classical signaling used by molecules with a PER-ARNT-SIM (PAS) domain such as AhR allows communication between the host and the external environment [30], which supports AhR’s initial characterization as a bioactivator and metabolizer of environmental toxins, xenobiotics, and carcinogens [2,31].

Ligand-free AhR resides in the cytoplasm complexed with a dimer of the heat shock protein 90 (HSP90), hepatitis B virus X-associated protein 2 (XAP-2; also known as the AhR-interacting protein (AIP)), c-SRC protein kinase, and p23 prior to ligand binding [32]. The first HSP90 molecule binds to the PAS region whereas the second interacts with both the bHLH region, an area involved in DNA binding, and the PAS region which is involved with ligand-binding [2,33]. AIP is involved in the stabilization of the chaperone complex and the AhR protein itself by preventing ubiquitination [34]. Upon ligand binding, this protein is released, and conformational changes occur that expose the nuclear localization signal [35]. The AhR translocates to the nucleus, but it is unclear which chaperone molecules join. Recent studies suggested that the translocation of HSP90 to the nucleus occurs upon activation with certain ligands [33,36]. This may be a ligand-specific determination, so further studies are needed.

**Figure 1:**
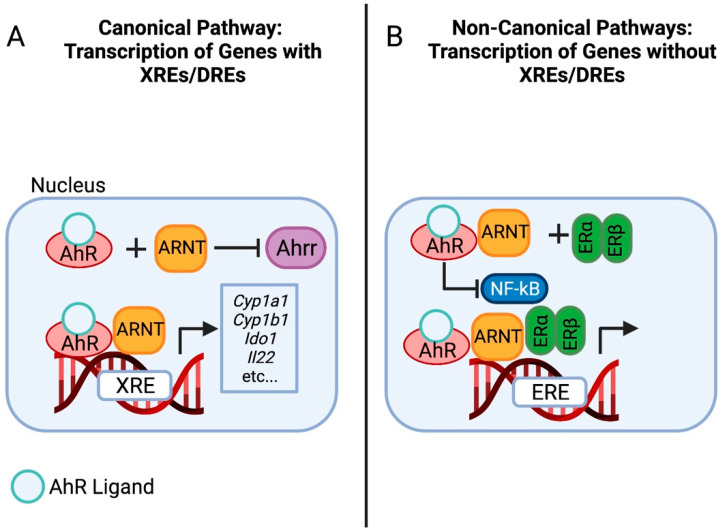
Canonical and Non-Canonical AhR Pathways. In the Canonical pathway, AhR activation by ligand leads to regulation of genes with XREs such as CYP enzymes among others. AhR also leads to transcription of genes without XREs, such as estrogen-responsive DNA elements, NK-κβ, and STAT proteins. (Created with BioRender.com on 17 November 2021).

With the help of its bHLH and PAS domains, AhR binds to the aryl hydrocarbon receptor nuclear translocator (ARNT; also known as HIF1ꞵ) in the nucleus to form a heterodimer [35,37,38,39]. This forms a DNA binding complex that activates one of the more well-characterized signaling pathways: the transcription of genes that contain xenobiotic responsive elements [XREs; also known as dioxin-responsive elements (DREs)] [39] (Figure 1A). As a result, AhR agonists are capable of inducing the expression of cytochrome P450 (CYP) enzymes along with many others [3,37,38,39,40].

In addition to regulating genes with XREs, AhR can be recruited to other target DNA sequences to regulate the expression of genes without XREs (Figure 1B). This has been observed in a uterine tumor cell line where AhR is recruited to estrogen-responsive DNA elements to induce transcriptional estrogenic effects upon complexing with receptors ER-α and ER-β [41]. AhR is also capable of regulating nuclear factor-κβ (NF-κβ) and signal transducer and activator of transcription (STAT) proteins, emphasizing the depth of its immunomodulatory effects [42,43,44].

In summary, AhR has a well-studied adaptive function that seems to be important in cellular defenses against exogenous toxins. Further, the physiological function of AhR has gained a wealth of attention more recently. Not only does it interact with signaling pathways involved in cell proliferation and cell cycle [45,46], cytokine secretion [47], cell adhesion and cell migration [48], but specifically in mammals, AhR seems to have been involved in the development and functions of the immune system and thus is present in a variety of immune cell types [35,37,49].

### 3.1. Different Types of Ligands

Since the ligand-binding site of the AhR is structurally flexible [50], many small molecules are able to act as ligands to activate it [35]. In addition to the exogenous ligands (2,3,7,8-tetrachlo-rodibenzo-p-dioxin (TCDD), benzo[a]pyrene, β-naphthoflavone, etc.), that have been examined extensively [51,52,53,54], many other endogenous and dietary AhR ligands have been identified and show differential properties upon ligand activation [55,56].

#### 3.1.1. Endogenous AhR Ligands

A physiological source of many AhR ligands comes from the metabolism of tryptophan primarily via the kynurenine pathway. In the context of inflammation, indoleamine 2,3-dioxygenase (IDO) and tryptophan 2,3-dioxygenase (TDO) are the two main enzymes that metabolize tryptophan and generate kynurenine [2]. Kynurenine is capable of activating AhR, and its production is stimulated via a positive feedback loop in that the expression of IDO1 is increased, leading to degradation of tryptophan and production of kynurenine [57]. This AhR agonist has been shown to increase the differentiation of Foxp3+ T regulatory cells (Tregs) [58]. Kynurenine also gives rise to other metabolites, such as kynurenic acid, that serve as potent AhR ligands [35].

6-formylindolo[3,2-b]carbazole (FICZ) is a high affinity agonist derived from oxidized tryptophan through ultraviolet radiation that stimulates AhR-mediated activation of CYP1A1, 1A2, and 1B1 [2,31]. It is ubiquitous in cell culture conditions and is rapidly metabolized in a CYP1A1 negative feedback loop [3]. This ligand has implications in genomic stability, circadian rhythms, and the immune response [35]. Additionally, FICZ has been shown to play an important role in barrier function. In the small intestine, FICZ treatment enhanced T cell release of IL-17 and IL-22 in the small intestine which induced a protective effect of the intestinal barriers of mice after ethanol and burn injury [59]. It also shows promise as a therapeutic due to its effects on intraepithelial lymphocytes [60].

Yet another endogenous AhR agonist produced from tryptophan metabolism is 2-(1′H-indole-3′-carbonyl)-thiazole-4-carboxylic acid methyl ester (ITE) [61]. It is a nontoxic AhR agonist that has been shown to act on both T cells and dendritic cells to suppress gut inflammation in colitis [62], as well as display anti-cancer activity in multiple cell types [63,64]. Additionally, it has been shown to induce G1/G0 cell cycle arrest and apoptosis in hepatocellular carcinoma cells as well as inhibit their migration [65]. Studies have shown that ITE enacts its effects in a similar mechanism to the toxic AhR ligand TCDD in that it suppresses the Th17 response and induces Tregs [66,67]. These data implicate its use as a therapy not only in inflammatory diseases but also against inflammation-driven cancers.

Research on tryptophan metabolites capable of activating AhR led to the identification of ligands generated by the microbiota. *Lactobacillus reuteri*, a microbe present throughout the gastrointestinal tract, has been shown to produce indole-3-aldehyde through the indole-pyruvate pathway [68]. Additional ligands microbially metabolized from indole have also been identified and implicated in barrier function [69]. If proven to limit intestinal inflammation and preserve the integrity of the gut barrier, these ligands may show promise in treating inflammatory diseases.

#### 3.1.2. Dietary AhR Ligands

Many ligands are also acquired from the host’s diet. One way this is accomplished is through the consumption of cruciferous vegetables. Indole-3-carbinol (I3C), a natural glucosinolate glucobrassicin metabolite, is converted into multiple derivatives through acid hydrolysis upon digestion and produces another agonist, 3,3′-diindolylmethane (DIM), as a major by-product [70,71]. These agonists have been shown to possess anti-inflammatory, as well as anti-cancer and antimicrobial properties [72,73,74]. Studies have implicated I3C in intestinal stem cell development and proposed regulatory mechanisms such as Wnt and Notch signalling [75,76]. Treatment with DIM has been shown to elicit protective effects against liver injury through suppression of reactive oxygen species, limiting the pro-inflammatory mediators and cytokines, and attenuating hepatocyte apoptosis [77].

The natural polyphenol resveratrol is found in many dietary sources, particularly plant products [78,79]. The anti-cancer effects of resveratrol have been well defined in various cell types [80,81], and extensively reviewed elsewhere [82,83]. In regard to its anti-inflammatory properties, our laboratory has shown that resveratrol elicits its immunological effects in allergic asthma by downregulating miR-34a to induce expression of Foxp3 [84]. Further, it has been implicated in the amelioration of colitis through alterations of gut microbiota towards microbial species that induce Tregs and suppress Th1/Th17 cells [85].

There are numerous dietary AhR ligands that have been reviewed in the recent past and thus, we have limited our discussion on these in the current review [37,86,87,88].

## 4. Mechanisms through Which AhR Activation Attenuates Inflammation

**Figure 2:**
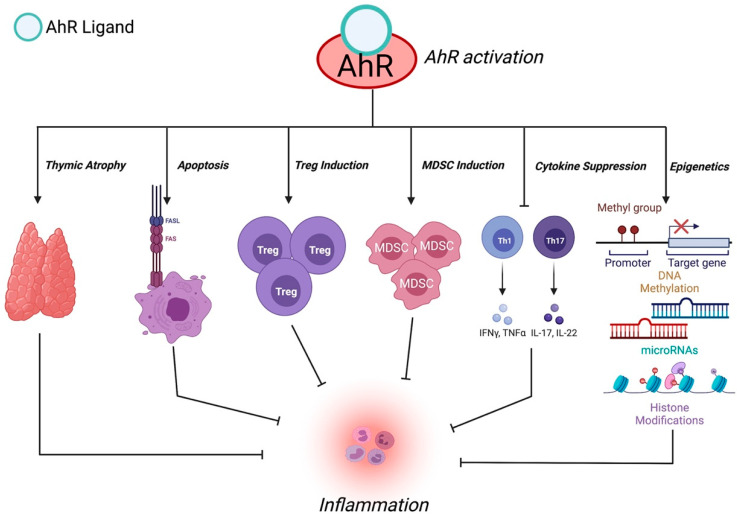
Mechanisms of Action of AhR Ligands to Suppress Inflammation (Created with BioRender.com on 17 November 2021).

In addition to regulating and inducing transcriptional changes, the AhR is capable of controlling cellular and molecular responses in a ligand-dependent manner [89]. Here, we address mechanisms used by AhR to mediate suppression of inflammation (Figure 2).

### 4.1. Thymic Atrophy

Thymic atrophy is a process that decreases the host’s ability to regenerate peripheral T cells resulting in disruption of thymic T cell development and differentiation. This process has been well defined in studies from the 1990s involving multiple animal species and TCDD [90]. A recent study has shown that TCDD-induced activation of AhR in dendritic cells is responsible for the observed thymic atrophy [91]. Further, supplementing nicotinamide adenine dinucleotide (NAD)+ through use of a form of vitamin B3 has been shown to prevent thymic atrophy, and thus reveals a role to combat TCDD-induced toxicity [92].

### 4.2. Apoptosis

In addition to AhR activation by TCDD leading to thymic atrophy, it has been reported that TCDD induces apoptosis [93]. Specifically, extensive studies have implicated Fas-Fas ligand (FasL) mediated apoptosis in activated T cells since it has been shown that FasL is upregulated upon AhR activation by TCDD leading to apoptosis [94,95]. Additionally, the promoter region of Fas contains a DRE with confirmed AhR binding, further implicating AhR in Fas-FasL interactions [93]. Furthermore, our laboratory has shown nuclear factor kappa B (NF-κB) and/or DREs with Fas-FasL regulation via Ahr due to multiple NF-kb motifs on the Fas and FasL promoters and presence of a DRE on the Fas promoter [96]. Of note, other mechanisms of apoptosis such as p53-mediated, have been associated with TCDD-induced activation of AhR [97].

### 4.3. Treg Induction

Anti-inflammatory T regulatory cells (Tregs) are known for their essential role in the conservation of tolerance to self-antigens as well as in the regulatory mechanisms of immune-mediated inflammation [98]. It has been shown in non-obese diabetic mice that neutralizing anti-IL-2 antibodies accelerate type 1 diabetes and that a reduction in IL-2 could impact Treg function and thus immune tolerance [99]. Taken together with the observance of raised anti-IL-2 antibodies in RA patients [100], loss of tolerance to IL-2 has been implicated as a mechanism through which autoimmune diseases are triggered due to its critical role in maintaining proper Treg function. The role of AhR in the activation of Tregs has been detailed in a recent review [101]. There are many different subsets of Tregs, but they are classically characterized as CD4 + CD25 + Foxp3+ and have been shown to contain higher expression levels of both AhR and CYP1A1 [102,103]. There have been many studies conducted that show that AhR activation by ligand is capable of increasing Tregs to reduce inflammation and ameliorate disease [62,66,71,85,103,104]. Recently, LPS-pretreated allogeneic hepatic stellate cells have been shown to use AhR-mediated mechanisms to expand and stabilize naturally occurring Tregs [105]. Together, these studies emphasize the role of AhR activation in Treg function to abate inflammation while also suggesting a critical role of IL-2 in T cell maintenance in autoimmunity.

Further, evidence suggests that CD4 + Foxp3− Tregs can be induced via AhR activation and are critical in reducing inflammation in murine models of inflammatory disease [101]. These were initially referred to as an AhR-dependent phenotype of cells that highly express CD25 and CTLA-4 [106]. Early in the graft versus host response, 10-chloro-7H-benzimidazo[2,1-a]benzo[de]Iso-quinolin-7-one (10-Cl-BBQ), a potent activator of AhR, induced these Foxp3− Tregs and suppressed effector cytotoxic T lymphocyte development [107]. Similar results were observed in cells treated with TCDD [108]. These studies show that AhR can induce the suppressive activity without Foxp3 expression identifying a novel mechanism of Treg induction.

### 4.4. MDSCs

Myeloid-derived suppressor cells (MDSCs) are a potent immunosuppressive cell type that has been associated with inhibition of T cell proliferation [109]. Our laboratory has shown that AhR activation by TCDD is capable of suppressing inflammation through induction of MDSCs [110]. This is accomplished by downregulation of miRNAs targeting anti-inflammatory and MDSC-regulatory genes leading to induction of chemokines and their receptors [110]. Specifically, CXCR2 has been implicated in the induction of MDSCs by TCDD [111]. Additionally, we have shown that microbiome dysbiosis plays a role in MDSC induction specifically in terms of observed increases in *Prevotella* and *Lactobacillus* and decreases in *Sutterella* and *Bacteroides* in TCDD-treated mice [111]. Together, these studies support the ability of AhR activation by TCDD to induce highly immunosuppressive cells of the myeloid lineage.

### 4.5. Cytokine Suppression

Cytokine suppression is in part responsible for the observed AhR-induced suppression of inflammatory states. Our laboratory has previously shown that TCDD-induced AhR activation employed DNA methylation mechanisms to reverse the demethylation of IL-17 promoters in colitis, thus inhibiting Th17 cells and attenuating inflammation [112]. In the intestine, AhR signaling in type 2 innate lymphoid cells (ILC2) suppressed expression of an IL-33 receptor (ST2) necessary for promotion of ILC2 responses as well as IL-5, IL-13 and amphiregulin [113]. Further, administration of FICZ was capable of ameliorating multiple models of colitis by employing mechanisms including the downregulation of inflammatory cytokines [114]. AhR’s ability to suppress pro-inflammatory cytokines is an essential mechanism, however, its effects are determined by the ligand used and the cell subsets involved.

### 4.6. Epigenetic Changes

Studies have provided evidence for AhR involvement in modulating chromatin remodeling through histone acetylation and methylation. Specifically, curcumin, another polyphenolic AhR ligand, has been shown to modulate the accessibility of DNA by inhibiting histone deacetylase (HDAC) activity at low concentrations and inhibiting histone acetyltransferase (HAT) activity at high concentrations [115]. Additionally, AhR-dependent mechanisms are involved in DNA methylation, histone modifications, and non-coding RNAs (ncRNAs) upon activation with TCDD, which has been reviewed elsewhere [54]. AhR has also been implicated in the control of long non-coding RNAs [116,117], microRNAs (miRNAs) [118], as well as others. Of note, miRNAs have also been shown to suppress AhR expression [104,119].

## 5. How Different Ligands Induce Different Immunological Changes

There is a great deal of work that supports the notion that many factors contribute to the effects observed upon AhR activation. Most importantly, (1) the characteristics of the ligand, (2) the cell type that expresses AhR, and (3) the coactivators present seem to be the main factors that determine the immunological outcome [120,121]. Additionally, other aspects of the microenvironment, such as the composition of the microbiota in the intestines and the presence or absence of differentiating cytokines, may influence the cell subset towards which AhR drives differentiation. Future studies should focus on the immunological changes induced upon activation with different ligands in the same inflammatory model, so that the differential mechanisms employed can be further elucidated.

Notably, recent studies have explored miRNA induction as a mechanism contributing to differential inflammatory responses. In a murine model of delayed-type hypersensitivity, it was shown that upregulation of miRNA-132 by TCDD led to induction of Tregs, while FICZ downregulated miRNA-132 expression and increased Th17 cells [122]. Similar findings have been shown in this model upon treatment with indoles I3C and DIM and their upregulation of miRNAs that target IL-17 (miRNA-495 and miRNA-1192) and downregulation of those that target Foxp3 (miRNA-31, miRNA-219, and miRNA-490) [71]. Thus, miRNA expression may play a role in inflammatory responses and AhR activation could represent a novel method to induce or suppress their expression.

## 6. AhR and Inflammatory Diseases

Considering the cellular and molecular mechanisms employed by AhR to regulate the immune response, it’s no surprise that activation of this receptor has shown promise in preventing or treating inflammatory diseases. While a host of disease models have been used to study effects (Table 1), we will discuss its implications in a select number of inflammatory diseases such as Inflammatory Bowel Diseases, Multiple Sclerosis, and skin conditions such as Atopic Dermatitis and Psoriasis.

### 6.1. Inflammatory Bowel Disease

Inflammatory bowel diseases, including ulcerative colitis and Crohn’s disease, are marked by chronic inflammation in the gastrointestinal tract and massive accumulation of leukocytes attempting to restrict pathogenic microorganisms [167]. Often, innate and T cell responses are dysregulated in these diseases due to a loss of homeostasis between genetic factors of the host and its gut microbiota often caused by an environmental trigger [168].

AhR activation by ligand has shown promise in protection against gut inflammation. Specifically, it was shown that I3C treatment suppresses colonic inflammation and prevents microbial dysbiosis through induction of IL-22 [145], a clinically relevant cytokine that has been shown to be involved in microbial host defenses and repair of the mucosa [169]. Studies show that immune tolerance promotion and suppression of IBD occurs through increased differentiation of Tregs. For example, our laboratory and others have shown that ITE induces Treg differentiation and thus, IL-10 production, as well as reduces the frequency of CD4 + Th17 cells and production of inflammatory cytokines [62,141].

As previously mentioned, gut dysbiosis plays an important role in the induction of IBD, therefore, manipulation of the gut microbiota has been considered as a therapy. While intestinal bacteria, such as strains of *Lactobacilli*, are capable of producing high levels AhR ligands, this ability is impacted in times of gut inflammation [170,171]. It has been shown that supplementation of *Lactobacilli* via inoculation results in a reduction of intestinal inflammation mediated through activation of AhR [172]. Additionally, ligands produced by *Lactobacilli* (such as indole-3-aldehyde) have been shown to induce transcription of *Il22* to provide antifungal resistance and mucosal protection from inflammation [173]. Together, these data implicate the microbiome in the production of ligands to affect AhR signaling and its larger role of maintaining homeostasis, suggesting microbiota manipulation as a beneficial therapeutic.

### 6.2. Multiple Sclerosis

Multiple sclerosis (MS) is a neurodegenerative disorder during which the myelin sheath surrounding the axon terminals are deemed immunogenic by the host’s immune system [174]. Encephalitogenic T helper cells have emerged as a primary driver of the inflammatory response and neurodegeneration in MS [175]. Additionally, as astrocytes are the most abundant in the brain, evidence has emerged implicating them in inflammatory responses in the central nervous system (CNS) upon activation by Th1 and Th17 effector cells [176,177]. Several AhR ligands including TCDD, indoles, resveratrol and the like have been shown in recent years to suppress experimental MS by attenuating neuroinflammation [103,150,178,179].

It is interesting to note that microbiota-derived AhR ligands can also serve as potent regulators of neuroinflammation, further characterizing the role for AhR in the gut-brain axis in suppressing MS progression. For example, a recent study suggested that tryptamine administration attenuates EAE by activating AhR and suppressing neuroinflammation [129]. Tryptamine treatment also caused alterations in the gut microbiota and promoted butyrate production [129]. Tryptophan metabolites produced by the intestinal microbiota along with type I interferons activate AhR in astrocytes and lead to suppression of inflammation [180]. Furthermore, AhR activation in microglia by indoxyl-3-sulfate (I3S) has been shown to suppress expression of pro-inflammatory and neurotoxic genes and increase *IL10* expression, which supports the capabilities of AhR ligands to limit CNS inflammation [181]. In addition, Urolithin A, an intestinal microbiota metabolic product, was shown to ameliorate experimental MS by reducing neuroinflammation caused by Th1 and Th17 cells [182]. The potential efficacy of AhR ligands in the treatment of clinical MS has been shown in a recent study in which Laquinimod, an AhR ligand, which was developed for the treatment of MS, was shown to attenuate experimental MS by mediating anti-inflammatory effects on glial cells [183,184].

Of note, an emerging concept exists where the role of a “top-down” mechanism in which brain health affects the microbiome has been suggested [185]. This brain-gut axis (BGA) concept suggests that urine can be used to detect CNS disorders in which bacterial pathogens cross the blood-brain barrier and induce changes in the gastrointestinal tract [185]. Further studies should be conducted to determine whether AhR activation on astrocytes and/or microglia induce changes in the gut microbiome.

### 6.3. Atopic Dermatitis and Psoriasis

Since AhR and ARNT are abundantly expressed in the skin, this complex has been targeted in the treatment of skin diseases such as atopic dermatitis (AD) and psoriasis. Both of these inflammatory conditions seem to involve pathogenic cytokine signaling, as evidenced by their response to current biologics [186], as well as infiltrating T cell and dendritic cell populations [187]. Currently, Tapinarof is a topical AhR agonist on the market used to treat both AD and psoriasis. It is structurally similar to resveratrol and has been shown to restore skin homeostasis by decreasing production of pro-inflammatory cytokines, activating the nuclear factor-erythroid 2-related factor-2 (NRF2) pathway to reduce oxidative stress, and increasing expression of skin barrier genes [188,189]. Additionally, FICZ, an endogenous AhR ligand reduced the inflammatory response in the imiquimod-induced model of skin inflammation [140].

## 7. Associations between AhR-Related Mutations in Humans and Inflammatory Diseases

Recent studies have shown that mutations affecting AhR activation play a role in inflammatory diseases. Caspase recruitment domain family member 9 (CARD9) has been implicated as one of many inflammatory bowel disease susceptibility genes [190]. Normally, it promotes activation of the IL-22 pathway to induce recovery from colitis. However, a reduced production of AhR ligands and AhR activation in individuals containing an IBD-associated single-nucleotide polymorphism (SNP) in CARD9 has been observed in those with IBD [172]. Additionally, pancreatic beta cell dysfunction leading to diabetes has been associated with ARNT due to an observed reduction in human islets from diabetic patients as well as the confirmed association of ARNT with hepatocyte nuclear factor 4α (HNF4α), a known mutated transcription factor in maturity-onset diabetes of the young (MODY) [191]. While specific AhR mutations are lacking, progress has been made towards identifying correlations between AhR, environmental factors, and inflammatory diseases [192]. Clearly additional studies are necessary to identify and understand mutations in AhR signaling pathways and its impact on human inflammatory diseases.

## 8. AhR Crystal Structure and Possibilities of Developing New Drugs

As previously described, ligand binding induces conformational changes in AhR exposing nuclear localization sequences [35]. AhR then forms a dimer with ARNT in the nucleus and is recruited to DREs [193]. Through use of molecular dynamic simulations (MDS), our laboratory has characterized the process of ligand binding to AhR. Results from this study indicated that some ligands, such as I3C and DIM, are less stable in the AhR ligand binding domain (AhRLBD) as compared to others, which may account for the differences in binding affinity with more stable ligands such as FICZ having higher affinity [121]. Studies have implicated this higher binding affinity with FICZ’s ability to activate AhR and induce immunological changes at a low dose as compared to a lower affinity ligand requiring doses that are not physiologically applicable [194]. Thus, it is possible that development of high affinity drugs to the AhRLBD will be more appropriate to induce immunological changes at a physiologically relevant dose without causing overt toxicity.

Additionally, the crystal structure of heterodimer forms of AhR have also been determined. Upon observation of both the AhR-ARNT heterodimer complexed with the DRE and the AhRR-ARNT heterodimer, the structures appeared highly intertwined and asymmetrical [193,195]. Similar to an inverted triangle, the two PAS domains of ARNT appeared at the top points, while the bHLH domains were positioned at the bottom with a centered PAS-A domain of AhRR [195]. Results also showed that a PAS-B region in the AhRR is lacking, thus allowing for the development of therapeutic strategies to limit excessive activation of AhR via AhRR repressive mechanisms [195].

Additional studies have suggested that the PAS-B domain of AhR (the ligand binding domain) is only needed for translocation to the nucleus, and thus, not necessary for heterodimerization [196]. Therefore, a purified AhR and ARNT complex containing only the PAS-A and bHLH domains bound to DNA was used and revealed three interaction interfaces that mediate stable dimerization. Induced mutations of the DNA binding residues present in these interfaces inhibited the function of the complex and hindered gene activation. Consequently, it has been suggested that targeting of these three assembly interfaces may be beneficial [197].

## 9. Concluding Remarks

Over the years, the aryl hydrocarbon receptor has been implicated in more diverse functions than originally believed. While it was considered to be the key environmental sensor regulating the toxicity of xenobiotics, there is an extensive amount of recent evidence that suggests its role in immune regulation, microbial defense, and barrier organ homeostasis suggesting this receptor as a beneficial target for the treatment and possible prevention of autoimmune and inflammatory diseases. Bacterial and viral infections also contribute to autoimmunity in that they could trigger the observed loss of tolerance via TLR signaling, IL-2 reduction or autoreactivity, amongst others. Considering the indispensable role of IL-2 in Treg function and AhR’s ability to induce Tregs, studies should explore the role of AhR to maintain IL-2 levels in such infections. While this receptor could prove to be a therapeutic option, there exists a dire need for more research and understanding of its many implications. One of the biggest concerns is that it is unclear how some AhR ligands such as TCDD can be highly toxic and carcinogenic, while other AhR ligands, especially the dietary as well as endogenous, are beneficial in maintaining immune system homeostasis. Secondly, it has been well established that the binding of ligands to AhR differs between species, so further studies should be conducted to determine whether the immunological changes induced upon ligand-activation of the receptor has species specificity as well. It is assumed that AhR is involved in immunomodulation, so it is imperative that studies are not only conducted in mouse cells, but also further established in human cells and humanized mouse models to increase their translational potential. In addition to species specificity, AhR also has cell-type specificity which introduces another challenge. The ability to control the tissue and cell-type in which AhR is activated is imperative to prevent off-target effects. A possible solution to this with promising results has emerged in the form of nanoparticle technology. Nanoparticles have been engineered to deliver ligands and other compounds in vivo to induce specific cell phenotypes and reestablish tolerance via AhR activation [198]. Together, the success of these studies would provide great promise for AhR as a therapeutic for immunomodulation.

## Figures and Tables

**Table 1:** Compounds that activate AhR and have been shown to be relevant in inflammatory disease.

Type of Ligand	Name of Ligand	Disease Implications
Xenobiotic	TCDD	*B. pertussis* infection [104], experimental autoimmune uveitis [123], multiple sclerosis [103], colitis [112,124], atopic dermatitis and psoriasis [55]
	β-naphthoflavone	Colitis [125], neuroinflammation [126], irradiation-induced intestinal injury [127]
Endogenous	Kynurenine Pathway Metabolites	Rheumatoid arthritis [128], multiple sclerosis [129,130,131], atherosclerosis [132], mastitis [133], rheumatoid arthritis [134]
	FICZ	Atopic dermatitis/psoriasis [55,122], acute kidney injury [135], lung fibrosis [136], colitis [137,138], periodontitis [139], skin inflammation [140]
	ITE	Colitis [62,141], cardiac repair [142], liver fibrosis [143], cancer [64,65,144]
Dietary	I3C	Colitis [145,146], retinal degenerative diseases [147], Parkinson’s Disease [148], systemic lupus erythematosus [149]
	DIM	Delayed-type hypersensitivity [71], multiple sclerosis [150,151], cancer [152], ischemia [153]
	Resveratrol	Immune thrombocytopenic purpura [154], Respiratory syncytial virus-mediated airway inflammation [155], acute respiratory distress syndrome [156], colitis [85], multiple sclerosis [157]
	Curcumin	Allergic asthma [158], colitis [159,160], multiple sclerosis [161], obesity [162], acute kidney injury [163], mastitis [164], non-alcoholic steatohepatitis [165], psoriasis [166]

## References

[1] Wheeler M.A., Rothhammer V., Quintana F.J. (2017). Control of immune-mediated pathology via the aryl hydrocarbon receptor. J. Biol. Chem..

[2] Stockinger B., Meglio P.D., Gialitakis M., Duarte J.H. (2014). The Aryl Hydrocarbon Receptor: Multitasking in the Immune System. Annu. Rev. Immunol..

[3] Esser C., Rannug A. (2015). The Aryl Hydrocarbon Receptor in Barrier Organ Physiology, Immunology, and Toxicology. Pharmacol. Rev..

[4] Medzhitov R. (2008). Origin and physiological roles of inflammation. Nature.

[5] Nathan C. (2002). Points of control in inflammation. Nature.

[6] Arulselvan P., Fard M.T., Tan W.S., Gothai S., Fakurazi S., Norhaizan M.E., Kumar S.S. (2016). Role of Antioxidants and Natural Products in Inflammation. Oxid. Med. Cell Longev..

[7] Takeuchi O., Akira S. (2010). Pattern Recognition Receptors and Inflammation. Cell.

[8] Iqbal A.J., Fisher E.A., Greaves D.R. (2016). Inflammation—A Critical Appreciation of the Role of Myeloid Cells. Microbiol. Spectr..

[9] Okin D., Medzhitov R. (2012). Evolution of Inflammatory Diseases. Curr. Biol..

[10] Feehan K.T., Gilroy D.W. (2019). Is Resolution the End of Inflammation?. Trends Mol. Med..

[11] Ospelt C., Brentano F., Rengel Y., Stanczyk J., Kolling C., Tak P.P., Gay R.E., Gay S., Kyburz D. (2008). Overexpression of toll-like receptors 3 and 4 in synovial tissue from patients with early rheumatoid arthritis: Toll-like receptor expression in early and longstanding arthritis. Arthritis Rheum..

[12] Huang Q., Ma Y., Adebayo A., Pope R.M. (2007). Increased macrophage activation mediated through toll-like receptors in rheumatoid arthritis. Arthritis Rheum..

[13] Kowalski M.L., Wolska A., Grzegorczyk J., Hilt J., Jarzebska M., Drobniewski M., Synder M., Kurowski M. (2008). Increased Responsiveness to Toll-Like Receptor 4 Stimulation in Peripheral Blood Mononuclear Cells from Patients with Recent Onset Rheumatoid Arthritis. Mediat. Inflamm..

[14] Farrugia M., Baron B. (2016). The role of TNF-α in rheumatoid arthritis: A focus on regulatory T cells. J. Clin. Transl. Res..

[15] Medzhitov R. (2010). Inflammation 2010: New Adventures of an Old Flame. Cell.

[16] Rose N.R. (2016). Prediction and Prevention of Autoimmune Disease in the 21st Century: A Review and Preview. Am. J. Epidemiol..

[17] Wang L., Wang F.-S., Gershwin M.E. (2015). Human autoimmune diseases: A comprehensive update. J. Intern. Med..

[18] Bo M., Niegowska M., Eames H.L., Almuttaqi H., Arru G., Erre G.L., Passiu G., Khoyratty T.E., van Grinsven E., Udalova I.A. (2020). Antibody response to homologous epitopes of Epstein-Barr virus, *Mycobacterium avium* subsp. paratuberculosis and IRF5 in patients with different connective tissue diseases and in mouse model of antigen-induced arthritis. J. Transl. Autoimmun..

[19] Bo M., Erre G.L., Niegowska M., Piras M., Taras L., Longu M.G., Passiu L.G., Sechi A. (2018). Interferon regulatory factor 5 is a potential target of autoimmune response triggered by Epstein-barr virus and Mycobacterium avium subsp. paratuberculosis in rheumatoid arthritis: Investigating a mechanism of molecular mimicry. Clin. Exp. Rheumatol..

[20] Langan D., Rose N.R., Moudgil K.D. (2020). Common innate pathways to autoimmune disease. Clin. Immunol..

[21] Arru G., Sechi E., Mariotto S., Zarbo I.R., Ferrari S., Gajofatto A., Monaco S., Deiana G.A., Bo M., Sechi L.A. (2019). Antibody response against HERV-W in patients with MOG-IgG associated disorders, multiple sclerosis and NMOSD. J. Neuroimmunol..

[22] Bo M., Niegowska M., Frau J., Sechi G., Arru G., Cocco E., Sechi L.A. (2020). IL-2 and Mycobacterial Lipoarabinomannan as Targets of Immune Responses in Multiple Sclerosis Patients. Microorganisms.

[23] Bo M., Niegowska M., Arru G., Sechi E., Mariotto S., Mancinelli C., Farinazzo A., Alberti D., Gajofatto A., Ferrari S. (2018). Mycobacterium avium subspecies paratuberculosis and myelin basic protein specific epitopes are highly recognized by sera from patients with *Neuromyelitis optica* spectrum disorder. J. Neuroimmunol..

[24] Arru G., Mameli G., Deiana G.A., Rassu A.L., Piredda R., Sechi E., Caggiu E., Bo M., Nako E., Urso D. (2018). Humoral immunity response to human endogenous retroviruses K/W differentiates between amyotrophic lateral sclerosis and other neurological diseases. Eur. J. Neurol..

[25] Arru G., Galleri G., Deiana G.A., Zarbo I.R., Sechi E., Bo M., Cadoni M.P.L., Corda D.G., Frau C., Simula E.R. (2021). HERV-K Modulates the Immune Response in ALS Patients. Microorganisms.

[26] Niegowska M., Wajda-Cuszlag M., Stępień-Ptak G., Trojanek J., Michałkiewicz J., Szalecki M., Sechi L.A. (2019). Anti-HERV-WEnv antibodies are correlated with seroreactivity against *Mycobacterium avium* subsp. paratuberculosis in children and youths at T1D risk. Sci. Rep..

[27] Mameli G., Erre G.L., Caggiu E., Mura S., Cossu D., Bo M., Cadoni M.L., Piras A., Mundula N., Colombo E. (2017). Identification of a HERV-K env surface peptide highly recognized in Rheumatoid Arthritis (RA) patients: A cross-sectional case–control study. Clin. Exp. Immunol..

[28] Cushing K., Higgins P.D.R. (2021). Management of Crohn Disease. JAMA.

[29] Hankinson O. (1995). The Aryl Hydrocarbon Receptor Complex. Annu. Rev. Pharmacol. Toxicol..

[30] Trikha P., Lee D.A. (2020). The role of AhR in transcriptional regulation of immune cell development and function. Biochim. Biophys. Acta (BBA)-Bioenergy.

[31] Mobini K., Banakar E., Tamaddon G., Mohammadi-Bardbori A. (2020). 6-Formylindolo[3,2-b]carbazole (FICZ) Enhances The Expression of Tumor Suppressor miRNAs, miR-22, miR-515-5p, and miR-124-3p in MCF-7 Cells. Cell J..

[32] Rothhammer V., Quintana F.J. (2019). The aryl hydrocarbon receptor: An environmental sensor integrating immune responses in health and disease. Nat. Rev. Immunol..

[33] Kudo I., Hosaka M., Haga A., Tsuji N., Nagata Y., Okada H., Fukuda K., Kakizaki Y., Okamoto T., Grave E. (2017). The regulation mechanisms of AhR by molecular chaperone complex. J. Biochem..

[34] Kazlauskas A., Poellinger L., Pongratz I. (2000). The Immunophilin-like Protein XAP2 Regulates Ubiquitination and Subcellular Localization of the Dioxin Receptor. J. Biol. Chem..

[35] Gutiérrez-Vázquez C., Quintana F.J. (2018). Regulation of the Immune Response by the Aryl Hydrocarbon Receptor. Immunity.

[36] Tsuji N., Fukuda K., Nagata Y., Okada H., Haga A., Hatakeyama S., Yoshida S., Okamoto T., Hosaka M., Sekine K. (2014). The activation mechanism of the aryl hydrocarbon receptor (AhR) by molecular chaperone HSP90. FEBS Open Bio.

[37] Busbee P.B., Rouse M., Nagarkatti M., Nagarkatti P.S. (2013). Use of natural AhR ligands as potential therapeutic modalities against inflammatory disorders. Nutr. Rev..

[38] Mimura J., Fujii-Kuriyama Y. (2003). Functional role of AhR in the expression of toxic effects by TCDD. Biochim. Biophys. Acta.

[39] Corre S., Tardif N., Mouchet N., LeClair H.M., Boussemart L., Gautron A., Bachelot L., Perrot A., Soshilov A., Rogiers A. (2018). Sustained activation of the Aryl hydrocarbon Receptor transcription factor promotes resistance to BRAF-inhibitors in melanoma. Nat. Commun..

[40] Esser C., Rannug A., Stockinger B. (2009). The aryl hydrocarbon receptor in immunity. Trends Immunol..

[41] Ohtake F., Takeyama K.-I., Matsumoto T., Kitagawa H., Yamamoto Y., Nohara K., Tohyama C., Krust A., Mimura J., Chambon P. (2003). Modulation of oestrogen receptor signalling by association with the activated dioxin receptor. Nature.

[42] Ishihara Y., Kado S.Y., Hoeper C., Harel S., Vogel C.F.A. (2019). Role of NF-kB RelB in Aryl Hydrocarbon Receptor-Mediated Ligand Specific Effects. Int. J. Mol. Sci..

[43] Kimura A., Naka T., Nohara K., Fujii-Kuriyama Y., Kishimoto T. (2008). Aryl hydrocarbon receptor regulates Stat1 activation and participates in the development of Th17 cells. Proc. Natl. Acad. Sci. USA.

[44] Vogel C.F.A., Sciullo E., Li W., Wong P., Lazennec G., Matsumura F. (2007). RelB, a New Partner of Aryl Hydrocarbon Receptor-Mediated Transcription. Mol. Endocrinol..

[45] Yin J., Sheng B., Han B., Pu A., Yang K., Li P., Wang Q., Xiao W., Yang H. (2016). The AhR is involved in the regulation of LoVo cell proliferation through cell cycle-associated proteins. Cell Biol. Int..

[46] Yin J., Sheng B., Qiu Y., Yang K., Xiao W., Yang H. (2016). Role of AhR in positive regulation of cell proliferation and survival. Cell Prolif..

[47] Tsai C.-H., Lee Y., Li C.-H., Cheng Y.-W., Kang J.-J. (2019). Down-regulation of aryl hydrocarbon receptor intensifies carcinogen-induced retinal lesion via SOCS3-STAT3 signaling. Cell Biol. Toxicol..

[48] Luo Y., Xu T., Xie H.Q., Guo Z., Zhang W., Chen Y., Sha R., Liu Y., Ma Y., Xu L. (2020). Effects of 2,3,7,8-tetrachlorodibenzo-p-dioxin on spontaneous movement of human neuroblastoma cells. Sci. Total Environ..

[49] Han B., Sheng B., Zhang Z., Pu A., Yin J., Wang Q., Yang K., Sun L., Yu M., Qiu Y. (2016). Aryl Hydrocarbon Receptor Activation in Intestinal Obstruction Ameliorates Intestinal Barrier Dysfunction via Suppression of MLCK-MLC Phosphorylation Pathway. Shock.

[50] Ho P.P., Steinman L. (2008). The aryl hydrocarbon receptor: A regulator of Th17 and Treg cell development in disease. Cell Res..

[51] Denison M.S., Nagy S.R. (2003). Activation of the Aryl Hydrocarbon Receptor by Structurally Diverse Exogenous and Endogenous Chemicals. Annu. Rev. Pharmacol. Toxicol..

[52] Segner H., Bailey C., Tafalla C., Bo J. (2021). Immunotoxicity of Xenobiotics in Fish: A Role for the Aryl Hydrocarbon Receptor (AhR)?. Int. J. Mol. Sci..

[53] Vogel C.F.A., Van Winkle L.S., Esser C., Haarmann-Stemmann T. (2020). The aryl hydrocarbon receptor as a target of environmental stressors—Implications for pollution mediated stress and inflammatory responses. Redox Biol..

[54] Patrizi B., De Cumis M.S. (2018). TCDD Toxicity Mediated by Epigenetic Mechanisms. Int. J. Mol. Sci..

[55] Um J.-Y., Kim H.B., Kang S.Y., Son J.H., Chung B.Y., Park C.W., Kim H.O. (2020). 2,3,7,8-Tetrachlorodibenzo-*p*-Dioxin Regulates the Expression of Aryl Hydrocarbon Receptor-Related Factors and Cytokines in Peripheral Blood Mononuclear Cells and CD4 + T cells from Patients with Atopic Dermatitis and Psoriasis. Ann. Dermatol..

[56] Abdulla O.A., Neamah W., Sultan M., Alghetaa H.K., Singh N., Busbee P.B., Nagarkatti M., Nagarkatti P. (2021). The Ability of AhR Ligands to Attenuate Delayed Type Hypersensitivity Reaction Is Associated with Alterations in the Gut Microbiota. Front. Immunol..

[57] Kaiser H., Parker E., Hamrick M.W. (2019). Kynurenine signaling through the aryl hydrocarbon receptor: Implications for aging and healthspan. Exp. Gerontol..

[58] Gabriely G., Wheeler M.A., Takenaka M.C., Quintana F.J. (2017). Role of AHR and HIF-1α in Glioblastoma Metabolism. Trends Endocrinol. Metab..

[59] Li X., Luck M.E., Hammer A.M., Cannon A.R., Choudhry M.A. (2020). 6-Formylindolo[3,2-b]carbazole (FICZ)–mediated protection of gut barrier is dependent on T cells in a mouse model of alcohol combined with burn injury. Biochim. Biophys. Acta (BBA)-Mol. Basis Dis..

[60] Zhang Z., Pu A., Yu M., Xiao W., Sun L., Cai Y., Yang H. (2019). Aryl hydrocarbon receptor activation modulates γδ intestinal intraepithelial lymphocytes and protects against ischemia/reperfusion injury in the murine small intestine. Mol. Med. Rep..

[61] Piwarski S.A., Thompson C., Chaudhry A.R., Denvir J., Primerano D.A., Fan J., Salisbury T.B. (2020). The putative endogenous AHR ligand ITE reduces JAG1 and associated NOTCH1 signaling in triple negative breast cancer cells. Biochem. Pharmacol..

[62] Abron J.D., Singh N.P., Mishra M., Price R.L., Nagarkatti M., Nagarkatti P.S., Singh U.P. (2018). An endogenous aryl hydrocarbon receptor ligand, ITE, induces regulatory T cells and ameliorates experimental colitis. Am. J. Physiol. Liver Physiol..

[63] Cheng J., Li W., Kang B., Zhou Y., Song J., Dan S., Yang Y., Zhang X., Li J., Yin S. (2015). Tryptophan derivatives regulate the transcription of Oct4 in stem-like cancer cells. Nat. Commun..

[64] Bian Y., Li Y., Shrestha G., Wen X., Cai B., Wang K., Wan X. (2019). ITE, an endogenous aryl hydrocarbon receptor ligand, suppresses endometrial cancer cell proliferation and migration. Toxicology.

[65] Zhang X., He B., Chen E., Lu J., Wang J., Cao H., Li L. (2020). The aryl hydrocarbon receptor ligand ITE inhibits cell proliferation and migration and enhances sensitivity to drug-resistance in hepatocellular carcinoma. J. Cell. Physiol..

[66] Quintana F.J., Murugaiyan G., Farez M.F., Mitsdoerffer M., Tukpah A.-M., Burns E.J., Weiner H.L. (2010). An endogenous aryl hydrocarbon receptor ligand acts on dendritic cells and T cells to suppress experimental autoimmune encephalomyelitis. Proc. Natl. Acad. Sci. USA.

[67] Zamali I., Rekik R., Hmida N.B., Ben Hmid A., Kammoun O., Barbouche M.-R., Ben Ahmed M. (2018). An endogenous aryl hydrocarbon receptor ligand enhances de novo generation of regulatory T cells in humans. J. Leukoc. Biol..

[68] Sun M., Ma N., He T., Johnston L.J., Ma X. (2019). Tryptophan (Trp) modulates gut homeostasis via aryl hydrocarbon receptor (AhR). Crit. Rev. Food Sci. Nutr..

[69] Scott S.A., Fu J., Chang P.V. (2020). Microbial tryptophan metabolites regulate gut barrier function via the aryl hydrocarbon receptor. Proc. Natl. Acad. Sci. USA.

[70] Wu Y., Li R.W., Huang H., Fletcher A., Yu L., Pham Q., Yu L., He Q., Wang T.T.Y. (2019). Inhibition of Tumor Growth by Dietary Indole-3-Carbinol in a Prostate Cancer Xenograft Model May Be Associated with Disrupted Gut Microbial Interactions. Nutrients.

[71] Singh N.P., Singh U.P., Rouse M., Zhang J., Chatterjee S., Nagarkatti P.S., Nagarkatti M. (2015). Dietary Indoles Suppress Delayed-Type Hypersensitivity by Inducing a Switch from Proinflammatory Th17 Cells to Anti-Inflammatory Regulatory T Cells through Regulation of MicroRNA. J. Immunol..

[72] Lee C.M., Park S.-H., Nam M.J. (2018). Anticarcinogenic effect of indole-3-carbinol (I3C) on human hepatocellular carcinoma SNU449 cells. Hum. Exp. Toxicol..

[73] Julliard W., De Wolfe T., Fechner J.H., Safdar N., Agni R., Mezrich J.D. (2017). Amelioration of Clostridium difficile Infection in Mice by Dietary Supplementation with Indole-3-carbinol. Ann. Surg..

[74] Du H., Zhang X., Zeng Y., Huang X., Chen H., Wang S., Wu J., Li Q., Zhu W., Li H. (2019). A Novel Phytochemical, DIM, Inhibits Proliferation, Migration, Invasion and TNF-α Induced Inflammatory Cytokine Production of Synovial Fibroblasts from Rheumatoid Arthritis Patients by Targeting MAPK and AKT/mTOR Signal Pathway. Front. Immunol..

[75] Park J.-H., Lee J.-M., Lee E.-J., Hwang W.-B., Kim D.-J. (2018). Indole-3-Carbinol Promotes Goblet-Cell Differentiation Regulating Wnt and Notch Signaling Pathways AhR-Dependently. Mol. Cells.

[76] Metidji A., Omenetti S., Crotta S., Li Y., Nye E., Ross E., Li V., Maradana M.R., Schiering C., Stockinger B. (2018). The Environmental Sensor AHR Protects from Inflammatory Damage by Maintaining Intestinal Stem Cell Homeostasis and Barrier Integrity. Immunity.

[77] Munakarmi S., Chand L., Shin H.B., Jang K.Y., Jeong Y.J. (2020). Indole-3-Carbinol Derivative DIM Mitigates Carbon Tetrachloride-Induced Acute Liver Injury in Mice by Inhibiting Inflammatory Response, Apoptosis and Regulating Oxidative Stress. Int. J. Mol. Sci..

[78] Ratz-Łyko A., Arct J. (2019). Resveratrol as an active ingredient for cosmetic and dermatological applications: A review. J. Cosmet. Laser Ther..

[79] Shin J.-W., Lee H.-S., Na J.-I., Huh C.-H., Park K.-C., Choi H.-R. (2020). Resveratrol Inhibits Particulate Matter-Induced Inflammatory Responses in Human Keratinocytes. Int. J. Mol. Sci..

[80] Khusbu F.Y., Zhou X., Roy M., Chen F.-Z., Cao Q., Chen H.-C. (2020). Resveratrol induces depletion of TRAF6 and suppresses prostate cancer cell proliferation and migration. Int. J. Biochem. Cell Biol..

[81] Qian W., Xiao Q., Wang L., Qin T., Xiao Y., Li J., Yue Y., Zhou C., Duan W., Ma Q. (2020). Resveratrol slows the tumourigenesis of pancreatic cancer by inhibiting NFκB activation. Biomed. Pharmacother..

[82] Berretta M., Bignucolo A., Di Francia R., Comello F., Facchini G., Ceccarelli M., Iaffaioli R.V., Quagliariello V., Maurea N. (2020). Resveratrol in Cancer Patients: From Bench to Bedside. Int. J. Mol. Sci..

[83] Kiskova T., Kubatka P., Büsselberg D., Kassayova M. (2020). The Plant-Derived Compound Resveratrol in Brain Cancer: A Review. Biomolecules.

[84] Alharris E., Alghetaa H., Seth R., Chatterjee S., Singh N.P., Nagarkatti M., Nagarkatti P. (2018). Resveratrol Attenuates Allergic Asthma and Associated Inflammation in the Lungs Through Regulation of miRNA-34a That Targets FoxP3 in Mice. Front. Immunol..

[85] Alrafas H.R., Busbee P.B., Nagarkatti M., Nagarkatti P.S. (2019). Resveratrol modulates the gut microbiota to prevent murine colitis development through induction of Tregs and suppression of Th17 cells. J. Leukoc. Biol..

[86] De Juan A., Segura E. (2021). Modulation of Immune Responses by Nutritional Ligands of Aryl Hydrocarbon Receptor. Front. Immunol..

[87] Han H., Safe S., Jayaraman A., Chapkin R.S. (2021). Diet–Host–Microbiota Interactions Shape Aryl Hydrocarbon Receptor Ligand Production to Modulate Intestinal Homeostasis. Annu. Rev. Nutr..

[88] Wisniewski P.J., Nagarkatti M., Nagarkatti P.S. (2021). Regulation of Intestinal Stem Cell Stemness by the Aryl Hydrocarbon Receptor and Its Ligands. Front. Immunol..

[89] Busbee P.B., Nagarkatti M., Nagarkatti P.S. (2015). Natural Indoles, Indole-3-Carbinol (I3C) and 3,3′-Diindolylmethane (DIM), Attenuate Staphylococcal Enterotoxin B-Mediated Liver Injury by Downregulating miR-31 Expression and Promoting Caspase-2-Mediated Apoptosis. PLoS ONE.

[90] Laiosa M.D., Wyman A., Murante F.G., Fiore N.C., Staples J.E., Gasiewicz T.A., Silverstone A.E. (2003). Cell proliferation arrest within intrathymic lymphocyte progenitor cells causes thymic atrophy mediated by the aryl hydrocarbon receptor. J. Immunol..

[91] Beamer C.A., Kreitinger J.M., Cole S.L., Shepherd D.M. (2019). Targeted deletion of the aryl hydrocarbon receptor in dendritic cells prevents thymic atrophy in response to dioxin. Arch. Toxicol..

[92] Diani-Moore S., Shoots J., Singh R., Zuk J.B., Rifkind A.B. (2017). NAD+ loss, a new player in AhR biology: Prevention of thymus atrophy and hepatosteatosis by NAD+ repletion. Sci. Rep..

[93] Fisher M.T., Nagarkatti M. (2004). Combined Screening of Thymocytes Using Apoptosis-Specific cDNA Array and Promoter Analysis Yields Novel Gene Targets Mediating TCDD-Induced Toxicity. Toxicol. Sci..

[94] Camacho I.A., Singh N., Hegde V.L., Nagarkatti M., Nagarkatti P.S. (2005). Treatment of Mice with 2,3,7,8-Tetrachlorodibenzo-p-Dioxin Leads to Aryl Hydrocarbon Receptor-Dependent Nuclear Translocation of NF-κB and Expression of Fas Ligand in Thymic Stromal Cells and Consequent Apoptosis in T Cells. J. Immunol..

[95] Fullerton A.M., Roth R.A., Ganey P.E. (2013). 2,3,7,8-TCDD enhances the sensitivity of mice to concanavalin A immune-mediated liver injury. Toxicol. Appl. Pharmacol..

[96] Singh N.P., Nagarkatti M., Nagarkatti P.S. (2007). Role of Dioxin Response Element and Nuclear Factor-κB Motifs in 2,3,7,8-Tetrachlorodibenzo-p-dioxin-Mediated Regulation of Fas and Fas Ligand Expression. Mol. Pharmacol..

[97] Das D.N., Panda P.K., Sinha N., Mukhopadhyay S., Naik P.P., Bhutia S.K. (2017). DNA damage by 2,3,7,8-tetrachlorodibenzo-p-dioxin-induced p53-mediated apoptosis through activation of cytochrome P450/aryl hydrocarbon receptor. Environ. Toxicol. Pharmacol..

[98] Sakaguchi S., Yamaguchi T., Nomura T., Ono M. (2008). Regulatory T Cells and Immune Tolerance. Cell.

[99] Pérol L., Lindner J.M., Caudana P., Nunez N.G., Baeyens A., Valle A., Sedlik C., Loirat D., Boyer O., Créange A. (2016). Loss of immune tolerance to IL-2 in type 1 diabetes. Nat. Commun..

[100] Bo M., Niegowska M., Erre G.L., Piras M., Longu M.G., Manchia P., Manca M., Passiu G., Sechi L.A. (2018). Rheumatoid arthritis patient antibodies highly recognize IL-2 in the immune response pathway involving IRF5 and EBV antigens. Sci. Rep..

[101] Singh N.P., Nagarkatti M., Nagarkatti P. (2020). From Suppressor T Cells to Regulatory T Cells: How the Journey that Began with the Discovery of the Toxic Effects of TCDD Led to Better Understanding of the Role of AhR in Immunoregulation. Int. J. Mol. Sci..

[102] Zou W. (2006). Regulatory T cells, tumour immunity and immunotherapy. Nat. Rev. Immunol..

[103] Quintana F.J., Basso A.S., Iglesias A.H., Korn T., Farez M.F., Bettelli E., Caccamo M., Oukka M., Weiner H.L. (2008). Control of T_reg_ and T_H_17 cell differentiation by the aryl hydrocarbon receptor. Nature.

[104] Al-Ghezi Z.Z., Singh N., Mehrpouya-Bahrami P., Busbee P.B., Nagarkatti M., Nagarkatti P.S. (2019). AhR Activation by TCDD (2,3,7,8-Tetrachlorodibenzo-p-dioxin) Attenuates Pertussis Toxin-Induced Inflammatory Responses by Differential Regulation of Tregs and Th17 Cells Through Specific Targeting by microRNA. Front. Microbiol..

[105] Kumar S., Wang J., Thomson A.W., Gandhi C.R. (2017). Hepatic stellate cells increase the immunosuppressive function of natural Foxp^3+^ regulatory T cells via IDO-induced AhR activation. J. Leukoc. Biol..

[106] Funatake C.J., Marshall N.B., Steppan L.B., Mourich D.V., Kerkvliet N.I. (2005). Cutting Edge: Activation of the Aryl Hydrocarbon Receptor by 2,3,7,8-Tetrachlorodibenzo-p-dioxin Generates a Population of CD4^+^ CD25^+^ Cells with Characteristics of Regulatory T Cells. J. Immunol..

[107] Punj S., Kopparapu P., Jang H.S., Phillips J.L., Pennington J., Rohlman D., O’Donnell E., Iversen P.L., Kolluri S.K., Kerkvliet N.I. (2014). Benzimidazoisoquinolines: A New Class of Rapidly Metabolized Aryl Hydrocarbon Receptor (AhR) Ligands that Induce AhR-Dependent Tregs and Prevent Murine Graft-Versus-Host Disease. PLoS ONE.

[108] Marshall N.B., Vorachek W.R., Steppan L.B., Mourich D.V., Kerkvliet N.I. (2008). Functional Characterization and Gene Expression Analysis of CD4^+^ CD25^+^ Regulatory T Cells Generated in Mice Treated with 2,3,7,8-Tetrachlorodibenzo-p-Dioxin. J. Immunol. Baltim..

[109] Kadhim S., Singh N.P., Zumbrun E.E., Cui T., Chatterjee S., Hofseth L., Abood A., Nagarkatti P., Nagarkatti M. (2018). Resveratrol-Mediated Attenuation of Staphylococcus aureus Enterotoxin B-Induced Acute Liver Injury Is Associated with Regulation of microRNA and Induction of Myeloid-Derived Suppressor Cells. Front. Microbiol..

[110] Neamah W.H., Singh N.P., Alghetaa H., Abdulla O., Chatterjee S., Busbee P.B., Nagarkatti M., Nagarkatti P. (2019). AhR Activation Leads to Massive Mobilization of Myeloid-Derived Suppressor Cells with Immunosuppressive Activity through Regulation of CXCR2 and MicroRNA miR-150-5p and miR-543-3p That Target Anti-Inflammatory Genes. J. Immunol..

[111] Neamah W.H., Busbee P.B., Alghetaa H., Abdulla O.A., Nagarkatti M., Nagarkatti P. (2020). AhR Activation Leads to Alterations in the Gut Microbiome with Consequent Effect on Induction of Myeloid Derived Suppressor Cells in a CXCR2-Dependent Manner. Int. J. Mol. Sci..

[112] Singh N.P., Singh U.P., Singh B., Price R.L., Nagarkatti M., Nagarkatti P.S. (2011). Activation of Aryl Hydrocarbon Receptor (AhR) Leads to Reciprocal Epigenetic Regulation of FoxP3 and IL-17 Expression and Amelioration of Experimental Colitis. PLoS ONE.

[113] Li S., Bostick J.W., Ye J., Qiu J., Zhang B., Urban J.F., Avram D., Zhou L. (2018). Aryl Hydrocarbon Receptor Signaling Cell Intrinsically Inhibits Intestinal Group 2 Innate Lymphoid Cell Function. Immunity.

[114] Monteleone I., Rizzo A., Sarra M., Sica G., Sileri P., Biancone L., Macdonald T.T., Pallone F., Monteleone G. (2011). Aryl Hydrocarbon Receptor-Induced Signals Up-regulate IL-22 Production and Inhibit Inflammation in the Gastrointestinal Tract. Gastroenterology.

[115] Mohammadi-Bardbori A., Akbarizadeh A.R., Delju F., Rannug A. (2016). Chromatin remodeling by curcumin alters endogenous aryl hydrocarbon receptor signaling. Chem. Interact..

[116] Grimaldi G., Rajendra S., Matthews J. (2018). The aryl hydrocarbon receptor regulates the expression of TIPARP and its cis long non-coding RNA, TIPARP-AS1. Biochem. Biophys. Res. Commun..

[117] Garcia G.R., Goodale B.C., Wiley M., La Du J.K., Hendrix D.A., Tanguay R.L. (2017). In Vivo Characterization of an AHR-Dependent Long Noncoding RNA Required for Proper Sox9b Expression. Mol. Pharmacol..

[118] Ushakov D.S., Dorozhkova A.S., Babayants E.V., Ovchinnikov V., Kushlinskii D.N., Adamyan L.V., Gulyaeva L.F., Kushlinskii N. (2018). Expression of microRNA Potentially Regulated by AhR and CAR in Malignant Tumors of the Endometrium. Bull. Exp. Biol. Med..

[119] Takenaka M.C., Gabriely G., Rothhammer V., Mascanfroni I.D., Wheeler M.A., Chao C.-C., Gutiérrez-Vázquez C., Kenison J., Tjon E.C., Barroso A. (2019). Control of tumor-associated macrophages and T cells in glioblastoma via AHR and CD39. Nat. Neurosci..

[120] Gargaro M., Pirro M., Romani R., Zelante T., Fallarino F. (2016). Aryl Hydrocarbon Receptor-Dependent Pathways in Immune Regulation. Arab. Archaeol. Epigr..

[121] Chitrala K.N., Yang X., Nagarkatti P., Nagarkatti M. (2018). Comparative analysis of interactions between aryl hydrocarbon receptor ligand binding domain with its ligands: A computational study. BMC Struct. Biol..

[122] Abdulla O.A., Neamah W., Sultan M., Chatterjee S., Singh N., Nagarkatti M., Nagarkatti P. (2021). AhR Ligands Differentially Regulate miRNA-132 Which Targets HMGB1 and to Control the Differentiation of Tregs and Th-17 Cells during Delayed-Type Hypersensitivity Response. Front. Immunol..

[123] Huang Y., He J., Liang H., Hu K., Jiang S., Yang L., Mei S., Zhu X., Yu J., Kijlstra A. (2018). Aryl Hydrocarbon Receptor Regulates Apoptosis and Inflammation in a Murine Model of Experimental Autoimmune Uveitis. Front. Immunol..

[124] Alzahrani A.M., Hanieh H., Ibrahim H.-I.M., Mohafez O., Shehata T., Ismail M.B., Alfwuaires M. (2017). Enhancing miR-132 expression by aryl hydrocarbon receptor attenuates tumorigenesis associated with chronic colitis. Int. Immunopharmacol..

[125] Furumatsu K., Nishiumi S., Kawano Y., Ooi M., Yoshie T., Shiomi Y., Kutsumi H., Ashida H., Fujii-Kuriyama Y., Azuma T. (2011). A Role of the Aryl Hydrocarbon Receptor in Attenuation of Colitis. Dig. Dis. Sci..

[126] Gao X., He D., Liu D., Hu G., Zhang Y., Meng T., Su Y., Zhou A., Huang B., Du J. (2020). Beta-naphthoflavone inhibits LPS-induced inflammation in BV-2 cells via AKT/Nrf-2/HO-1-NF-κB signaling axis. Immunobiology.

[127] Zhou X., Li D., Xu W., Zhang H., Wang H., Perdew G.H. (2020). β-Naphthoflavone Activation of the Ah Receptor Alleviates Irradiation-Induced Intestinal Injury in Mice. Antioxidants.

[128] Criado G., Šimelyte E., Inglis J.J., Essex D., Williams R.O. (2009). Indoleamine 2,3 dioxygenase-mediated tryptophan catabolism regulates accumulation of Th1/Th17 cells in the joint in collagen-induced arthritis. Arthritis Rheum..

[129] Dopkins N., Becker W., Miranda K., Walla M., Nagarkatti P., Nagarkatti M. (2021). Tryptamine Attenuates Experimental Multiple Sclerosis through Activation of Aryl Hydrocarbon Receptor. Front. Pharmacol..

[130] Zarzecki M.S., Souza L.C., Giacomeli R., Silva M.R.P., Prigol M., Boeira S.P., Jesse C.R. (2020). Involvement of Indoleamine-2,3-Dioxygenase and Kynurenine Pathway in Experimental Autoimmune Encephalomyelitis in Mice. Neurochem. Res..

[131] Sundaram G., Lim E., Brew B.J., Guillemin G.J. (2020). Kynurenine pathway modulation reverses the experimental autoimmune encephalomyelitis mouse disease progression. J. Neuroinflamm..

[132] Forteza M.J., Polyzos K.A., Baumgartner R., Suur B.E., Mussbacher M., Johansson D.K., Hermansson A., Hansson G.K., Ketelhuth D.F.J. (2018). Activation of the Regulatory T-Cell/Indoleamine 2,3-Dioxygenase Axis Reduces Vascular Inflammation and Atherosclerosis in Hyperlipidemic Mice. Front. Immunol..

[133] Zhao C., Wu K., Bao L., Chen L., Feng L., Liu Z., Wang Y., Fu Y., Zhang N., Hu X. (2021). Kynurenic acid protects against mastitis in mice by ameliorating inflammatory responses and enhancing blood-milk barrier integrity. Mol. Immunol..

[134] Balog A., Varga B., Fülöp F., Lantos I., Toldi G., Vécsei L., Mándi Y. (2021). Kynurenic Acid Analog Attenuates the Production of Tumor Necrosis Factor-α, Calgranulins (S100A 8/9 and S100A 12), and the Secretion of HNP1–3 and Stimulates the Production of Tumor Necrosis Factor-Stimulated Gene-6 in Whole Blood Cultures of Patients with Rheumatoid Arthritis. Front. Immunol..

[135] Tao S., Guo F., Ren Q., Liu J., Wei T., Li L., Ma L., Fu P. (2021). Activation of aryl hydrocarbon receptor by 6-formylindolo[3,2-b]carbazole alleviated acute kidney injury by repressing inflammation and apoptosis. J. Cell. Mol. Med..

[136] Takei H., Yasuoka H., Yoshimoto K., Takeuchi T. (2020). Aryl hydrocarbon receptor signals attenuate lung fibrosis in the bleomycin-induced mouse model for pulmonary fibrosis through increase of regulatory T cells. Arthritis Res..

[137] Yu K., Ma Y., Zhang Z., Fan X., Li T., Li L., Xiao W., Cai Y., Sun L., Xu P. (2018). AhR activation protects intestinal epithelial barrier function through regulation of Par-6. J. Mol. Histol..

[138] Ma Y., Wang Q., Yu K., Fan X., Xiao W., Cai Y., Xu P., Yu M., Yang H. (2018). 6-Formylindolo[3,2-b]carbazole induced aryl hydrocarbon receptor activation prevents intestinal barrier dysfunction through regulation of claudin-2 expression. Chem. Interact..

[139] Huang J., Cai X., Ou Y., Fan L., Zhou Y., Wang Y. (2019). Protective roles of FICZ and aryl hydrocarbon receptor axis on alveolar bone loss and inflammation in experimental periodontitis. J. Clin. Periodontol..

[140] Di Meglio P., Duarte J.H., Ahlfors H., Owens N.D., Li Y., Villanova F., Tosi I., Hirota K., Nestle F.O., Mrowietz U. (2014). Activation of the Aryl Hydrocarbon Receptor Dampens the Severity of Inflammatory Skin Conditions. Immunity.

[141] Goettel J.A., Gandhi R., Kenison J., Yeste A., Murugaiyan G., Sambanthamoorthy S., Griffith A.E., Patel B., Shouval D.S., Weiner H.L. (2016). AHR Activation Is Protective against Colitis Driven by T Cells in Humanized Mice. Cell Rep..

[142] Seong E., Lee J., Lim S., Park E., Kim E., Kim C.W., Lee E., Oh G., Choo E.H., Hwang B. (2021). Activation of Aryl Hydrocarbon Receptor by ITE Improves Cardiac Function in Mice after Myocardial Infarction. J. Am. Heart Assoc..

[143] Yan J., Tung H.-C., Li S., Niu Y., Garbacz W.G., Lu P., Bi Y., Li Y., He J., Xu M. (2019). Aryl Hydrocarbon Receptor Signaling Prevents Activation of Hepatic Stellate Cells and Liver Fibrogenesis in Mice. Gastroenterology.

[144] Zhao L., Shu Q., Sun H., Ma Y., Kang D., Zhao Y., Lu J., Gong P., Yang F., Wan F. (2020). 1′H-Indole-3′-Carbonyl-Thiazole-4-Carboxylic Acid Methyl Ester Blocked Human Glioma Cell Invasion via Aryl Hydrocarbon Receptor’s Regulation of Cytoskeletal Contraction. BioMed Res. Int..

[145] Busbee P.B., Menzel L., Alrafas H.R., Dopkins N., Becker W., Miranda K., Tang C., Chatterjee S., Singh U.P., Nagarkatti M. (2020). Indole-3-carbinol prevents colitis and associated microbial dysbiosis in an IL-22–dependent manner. JCI Insight.

[146] Riemschneider S., Hoffmann M., Slanina U., Weber K., Hauschildt S., Lehmann J. (2021). Indol-3-Carbinol and Quercetin Ameliorate Chronic DSS-Induced Colitis in C57BL/6 Mice by AhR-Mediated Anti-Inflammatory Mechanisms. Int. J. Environ. Res. Public Health.

[147] Khan A.S., Langmann T. (2020). Indole-3-carbinol regulates microglia homeostasis and protects the retina from degeneration. J. Neuroinflamm..

[148] Saini N., Akhtar A., Chauhan M., Dhingra N., Sah S.P. (2020). Protective effect of Indole-3-carbinol, an NF-κB inhibitor in experimental paradigm of Parkinson’s disease: In silico and in vivo studies. Brain Behav. Immun..

[149] Mohammadi S., Memarian A., Sedighi S., Behnampour N., Yazdani Y. (2018). Immunoregulatory effects of indole-3-carbinol on monocyte-derived macrophages in systemic lupus erythematosus: A crucial role for aryl hydrocarbon receptor. Autoimmunity.

[150] Rouse M., Singh N.P., Nagarkatti P.S., Nagarkatti M. (2013). Indoles mitigate the development of experimental autoimmune encephalomyelitis by induction of reciprocal differentiation of regulatory T cells and Th17 cells. Br. J. Pharmacol..

[151] Yang S., Tan L., Chen Y., Liu A., Hong M., Peng Z. (2020). DIM mitigates the development of experimental autoimmune encephalomyelitis by maintaining the stability and suppressive function of regulatory T cells. Cell. Immunol..

[152] Zhu P., Zhou K., Lu S., Bai Y., Qi R., Zhang S. (2020). Modulation of aryl hydrocarbon receptor inhibits esophageal squamous cell carcinoma progression by repressing COX_2_/PGE_2_/STAT_3_ axis. J. Cell Commun. Signal..

[153] Rzemieniec J., Wnuk A., Lasoń W., Bilecki W., Kajta M. (2019). The neuroprotective action of 3,3′-diindolylmethane against ischemia involves an inhibition of apoptosis and autophagy that depends on HDAC and AhR/CYP1A1 but not ERα/CYP_19_A1 signaling. Apoptosis.

[154] Guo N.-H., Fu X., Zi F.-M., Song Y., Wang S., Cheng J. (2019). The potential therapeutic benefit of resveratrol on Th17/Treg imbalance in immune thrombocytopenic purpura. Int. Immunopharmacol..

[155] Zang N., Xie X., Deng Y., Wu S., Wang L., Peng C., Li S., Ni K., Luo Y., Liu E. (2011). Resveratrol-Mediated Gamma Interferon Reduction Prevents Airway Inflammation and Airway Hyperresponsiveness in Respiratory Syncytial Virus-Infected Immunocompromised Mice. J. Virol..

[156] Alghetaa H., Mohammed A., Zhou J., Singh N., Nagarkatti M., Nagarkatti P. (2021). Resveratrol-mediated attenuation of superantigen-driven acute respiratory distress syndrome is mediated by microbiota in the lungs and gut. Pharmacol. Res..

[157] Gandy K.A.O., Zhang J., Nagarkatti P., Nagarkatti M. (2019). Resveratrol (3,5,4′-Trihydroxy-trans-Stilbene) Attenuates a Mouse Model of Multiple Sclerosis by Altering the miR-124/Sphingosine Kinase 1 Axis in Encephalitogenic T Cells in the Brain. J. Neuroimmune Pharmacol..

[158] Islam R., Dash D., Singh R. (2022). Intranasal curcumin and sodium butyrate modulates airway inflammation and fibrosis via HDAC inhibition in allergic asthma. Cytokine.

[159] Kang Z.-P., Wang M.-X., Wu T.-T., Liu D.-Y., Wang H.-Y., Long J., Zhao H.-M., Zhong Y.-B. (2021). Curcumin Alleviated Dextran Sulfate Sodium-Induced Colitis by Regulating M1/M2 Macrophage Polarization and TLRs Signaling Pathway. Evid. -Based Complement. Altern. Med..

[160] Zhong Y.-B., Kang Z.-P., Zhou B.-G., Wang H.-Y., Long J., Zhou W., Zhao H.-M., Liu D.-Y. (2021). Curcumin Regulated the Homeostasis of Memory T Cell and Ameliorated Dextran Sulfate Sodium-Induced Experimental Colitis. Front. Pharmacol..

[161] Mavaddatiyan L., Khezri S., Froushani S.M.A. (2021). Molecular effects of curcumin on the experimental autoimmune encephalomyelitis. Vet. Res. Forum.

[162] Islam T., Koboziev I., Albracht-Schulte K., Mistretta B., Scoggin S., Yosofvand M., Moussa H., Zabet-Moghaddam M., Ramalingam L., Gunaratne P.H. (2021). Curcumin Reduces Adipose Tissue Inflammation and Alters Gut Microbiota in Diet-Induced Obese Male Mice. Mol. Nutr. Food Res..

[163] Li L., Liu S., Zhou Y., Zhao M., Wang Y., Wang C., Lou P., Huang R., Ma L., Lu Y. (2021). Indispensable role of mitochondria in maintaining the therapeutic potential of curcumin in acute kidney injury. J. Cell. Mol. Med..

[164] Lebda M.A., Elmassry I.H., Taha N.M., Elfeky M.S. (2021). Nanocurcumin alleviates inflammation and oxidative stress in LPS-induced mastitis via activation of Nrf2 and suppressing TLR_4_-mediated NF-κB and HMGB1 signaling pathways in rats. Environ. Sci. Pollut. Res..

[165] Tong C., Wu H., Gu D., Li Y., Fan Y., Zeng J., Ding W. (2021). Effect of curcumin on the non-alcoholic steatohepatitis via inhibiting the M1 polarization of macrophages. Hum. Exp. Toxicol..

[166] Zhou T., Zhang S., Zhou Y., Lai S., Chen Y., Geng Y., Wang J. (2021). Curcumin alleviates imiquimod-induced psoriasis in progranulin-knockout mice. Eur. J. Pharmacol..

[167] Pernomian L., Duarte-Silva M., Cardoso C.R.D.B. (2020). The Aryl Hydrocarbon Receptor (AHR) as a Potential Target for the Control of Intestinal Inflammation: Insights from an Immune and Bacteria Sensor Receptor. Clin. Rev. Allergy Immunol..

[168] Larabi A., Barnich N., Nguyen H.T.T. (2019). New insights into the interplay between autophagy, gut microbiota and inflammatory responses in IBD. Autophagy.

[169] Mizoguchi A., Yano A., Himuro H., Ezaki Y., Sadanaga T., Mizoguchi E. (2018). Clinical importance of IL-22 cascade in IBD. J. Gastroenterol..

[170] Krishnan S., Ding Y., Saedi N., Choi M., Sridharan G.V., Sherr D.H., Yarmush M.L., Alaniz R.C., Jayaraman A., Lee K. (2018). Gut Microbiota-Derived Tryptophan Metabolites Modulate Inflammatory Response in Hepatocytes and Macrophages. Cell Rep..

[171] Natividad J.M., Agus A., Planchais J., Lamas B., Jarry A.C., Martin R., Michel M.-L., Chong-Nguyen C., Roussel R., Straube M. (2018). Impaired Aryl Hydrocarbon Receptor Ligand Production by the Gut Microbiota Is a Key Factor in Metabolic Syndrome. Cell Metab..

[172] Lamas B., Richard M.L., Leducq V., Pham H.-P., Michel M.-L., DA Costa G., Bridonneau C., Jegou S., Hoffmann T.W., Natividad J.M. (2016). CARD9 impacts colitis by altering gut microbiota metabolism of tryptophan into aryl hydrocarbon receptor ligands. Nat. Med..

[173] Zelante T., Iannitti R.G., Cunha C., De Luca A., Giovannini G., Pieraccini G., Zecchi R., D’Angelo C., Massi-Benedetti C., Fallarino F. (2013). Tryptophan catabolites from microbiota engage aryl hydrocarbon receptor and balance mucosal reactivity via interleukin-22. Immunity.

[174] Dopkins N., Nagarkatti P.S., Nagarkatti M. (2018). The role of gut microbiome and associated metabolome in the regulation of neuroinflammation in multiple sclerosis and its implications in attenuating chronic inflammation in other inflammatory and autoimmune disorders. Immunology.

[175] Kunkl M., Frascolla S., Amormino C., Volpe E., Tuosto L. (2020). T Helper Cells: The Modulators of Inflammation in Multiple Sclerosis. Cells.

[176] Prajeeth C.K., Kronisch J., Khorooshi R., Knier B., Toft-Hansen H., Gudi V., Floess S., Huehn J., Owens T., Korn T. (2017). Effectors of Th_1_ and Th_17_ cells act on astrocytes and augment their neuroinflammatory properties. J. Neuroinflamm..

[177] Brambilla R. (2019). The contribution of astrocytes to the neuroinflammatory response in multiple sclerosis and experimental autoimmune encephalomyelitis. Acta Neuropathol..

[178] Singh N.P., Hegde V.L., Hofseth L.J., Nagarkatti M., Nagarkatti P. (2007). Resveratrol (trans-3,5,4′-Trihydroxystilbene) Ameliorates Experimental Allergic Encephalomyelitis, Primarily via Induction of Apoptosis in T Cells Involving Activation of Aryl Hydrocarbon Receptor and Estrogen Receptor. Mol. Pharmacol..

[179] Yang E.-J., Stokes J.V., Kummari E., Eells J., Kaplan B.L. (2016). Immunomodulation By Subchronic Low Dose 2,3,7,8-Tetrachlorodibenzo-p-Dioxin in Experimental Autoimmune Encephalomyelitis in the Absence of Pertussis Toxin. Toxicol. Sci..

[180] Rothhammer V., Mascanfroni I.D., Bunse L., Takenaka M.C., Kenison J., Mayo L., Chao C.-C., Patel B., Yan R., Blain M. (2016). Type I interferons and microbial metabolites of tryptophan modulate astrocyte activity and central nervous system inflammation via the aryl hydrocarbon receptor. Nat. Med..

[181] Rothhammer V., Borucki D.M., Tjon E.C., Takenaka M.C., Chao C.-C., Ardura-Fabregat A., de Lima K.A., Gutiérrez-Vázquez C., Hewson P., Staszewski O. (2018). Microglial control of astrocytes in response to microbial metabolites. Nature.

[182] Shen P.-X., Li X., Deng S.-Y., Zhao L., Zhang Y.-Y., Deng X., Han B., Yu J., Li Y., Wang Z.-Z. (2021). Urolithin A ameliorates experimental autoimmune encephalomyelitis by targeting aryl hydrocarbon receptor. EBioMedicine.

[183] Rothhammer V., Kenison J.E., Li Z., Tjon E., Takenaka M.C., Chao C.-C., de Lima K.A., Borucki D.M., Kaye J., Quintana F.J. (2021). Aryl Hydrocarbon Receptor Activation in Astrocytes by Laquinimod Ameliorates Autoimmune Inflammation in the CNS. Neurol.-Neuroimmunol. Neuroinflamm..

[184] Kaye J., Piryatinsky V., Birnberg T., Hingaly T., Raymond E., Kashi R., Amit-Romach E., Caballero I.S., Towfic F., Ator M.A. (2016). Laquinimod arrests experimental autoimmune encephalomyelitis by activating the aryl hydrocarbon receptor. Proc. Natl. Acad. Sci. USA.

[185] Isaiah S., Loots D.T., Solomons R., Van Der Kuip M., Van Furth A.M.T., Mason S. (2020). Overview of Brain-to-Gut Axis Exposed to Chronic CNS Bacterial Infection(s) and a Predictive Urinary Metabolic Profile of a Brain Infected by Mycobacterium tuberculosis. Front. Neurosci..

[186] Furue M., Hashimoto-Hachiya A., Tsuji G. (2019). Aryl Hydrocarbon Receptor in Atopic Dermatitis and Psoriasis. Int. J. Mol. Sci..

[187] Werfel T., Allam J.-P., Biedermann T., Eyerich K., Gilles S., Guttman-Yassky E., Hoetzenecker W., Knol E., Simon H.-U., Wollenberg A. (2016). Cellular and molecular immunologic mechanisms in patients with atopic dermatitis. J. Allergy Clin. Immunol..

[188] Paller A.S., Gold L.S., Soung J., Tallman A.M., Rubenstein D.S., Gooderham M. (2020). Efficacy and patient-reported outcomes from a phase 2b, randomized clinical trial of tapinarof cream for the treatment of adolescents and adults with atopic dermatitis. J. Am. Acad. Dermatol..

[189] Smith S.H., Jayawickreme C., Rickard D.J., Nicodeme E., Bui T., Simmons C., Coquery C.M., Neil J., Pryor W.M., Mayhew D. (2017). Tapinarof Is a Natural AhR Agonist that Resolves Skin Inflammation in Mice and Humans. J. Investig. Dermatol..

[190] Hartjes L., Ruland J. (2019). CARD9 Signaling in Intestinal Immune Homeostasis and Oncogenesis. Front. Immunol..

[191] Gunton J.E., Kulkarni R.N., Yim S., Okada T., Hawthorne W.J., Tseng Y.-H., Roberson R.S., Ricordi C., O’Connell P.J., Gonzalez F.J. (2005). Loss of ARNT/HIF1β Mediates Altered Gene Expression and Pancreatic-Islet Dysfunction in Human Type 2 Diabetes. Cell.

[192] Fu J., Nogueira S.V., van Drongelen V., Coit P., Ling S., Rosloniec E.F., Sawalha A.H., Holoshitz J. (2018). Shared epitope–aryl hydrocarbon receptor crosstalk underlies the mechanism of gene–environment interaction in autoimmune arthritis. Proc. Natl. Acad. Sci. USA.

[193] Seok S.-H., Lee W., Jiang L., Molugu K., Zheng A., Li Y., Park S., Bradfield C.A., Xing Y. (2017). Structural hierarchy controlling dimerization and target DNA recognition in the AHR transcriptional complex. Proc. Natl. Acad. Sci. USA.

[194] Veldhoen M., Hirota K., Westendorf A.M., Buer J., Dumoutier L., Renauld J.C., Stockinger B. (2008). The aryl hydrocarbon receptor links TH17-cell-mediated autoimmunity to environmental toxins. Nature.

[195] Sakurai S., Shimizu T., Ohto U. (2017). The crystal structure of the AhRR–ARNT heterodimer reveals the structural basis of the repression of AhR-mediated transcription. J. Biol. Chem..

[196] Andersson P., McGuire J., Rubio C., Gradin K., Whitelaw M.L., Pettersson S., Hanberg A., Poellinger L. (2002). A constitutively active dioxin/aryl hydrocarbon receptor induces stomach tumors. Proc. Natl. Acad. Sci. USA.

[197] Schulte K.W., Green E., Wilz A., Platten M., Daumke O. (2017). Structural Basis for Aryl Hydrocarbon Receptor-Mediated Gene Activation. Structure.

[198] Yeste A., Takenaka M.C., Mascanfroni I.D., Nadeau M., Kenison J.E., Patel B., Tukpah A.-M., Babon J.A.B., DeNicola M., Kent S.C. (2016). Tolerogenic nanoparticles inhibit T cell–mediated autoimmunity through SOCS2. Sci. Signal..

